# Developmental Dynamics of Rett Syndrome

**DOI:** 10.1155/2016/6154080

**Published:** 2016-01-31

**Authors:** Danielle Feldman, Abhishek Banerjee, Mriganka Sur

**Affiliations:** ^1^Picower Institute for Learning and Memory, Department of Brain and Cognitive Sciences, Massachusetts Institute of Technology, Cambridge, MA 02139, USA; ^2^Laboratory of Neural Circuit Dynamics, Brain Research Institute, University of Zurich, 8057 Zurich, Switzerland

## Abstract

Rett Syndrome was long considered to be simply a disorder of postnatal development, with phenotypes that manifest only late in development and into adulthood. A variety of recent evidence demonstrates that the phenotypes of Rett Syndrome are present at the earliest stages of brain development, including developmental stages that define neurogenesis, migration, and patterning in addition to stages of synaptic and circuit development and plasticity. These phenotypes arise from the pleotropic effects of MeCP2, which is expressed very early in neuronal progenitors and continues to be expressed into adulthood. The effects of MeCP2 are mediated by diverse signaling, transcriptional, and epigenetic mechanisms. Attempts to reverse the effects of Rett Syndrome need to take into account the developmental dynamics and temporal impact of MeCP2 loss.

## 1. Introduction

Rett Syndrome (RTT) is a developmental neurological disorder that affects 1 in every 10,000–15,000 live births in the US [1]. The genetic origin of RTT, in ~90% of patients, has been traced to sporadic loss-of-function mutations in the X-linked gene* MECP2* coding for methyl CpG-binding protein 2, mainly localized to methylated pericentric heterochromatin [2]. Clinical features of the disorder involve marked developmental regression, progressive loss of acquired motor and language skills, the acquisition of stereotyped repetitive hand movements, muscle hypotonia, autonomic dysfunctions, and severe cognitive impairment.

MeCP2 is an epigenetic modulator of gene expression. It acts as both a transcriptional activator and repressor [3, 4], in addition to regulating gene expression posttranscriptionally via microRNA- (miRNA-) processing machinery [5] and in an activity-dependent manner to regulate synaptic activity [6]. The binding interaction between MeCP2 and DNA is governed by a variety of genetic and epigenetic factors such as the length of the DNA, nearby sequences, and methylation patterns [3, 7, 8]. MeCP2 is a known binding partner of 5-methylcytosine (5mC) at CpG dinucleotides throughout the genome, resulting in transcriptional repression in these regions [9]. However, MeCP2 is also the predominant 5-hydroxymethylcytosine- (5hmC-) binding protein in the brain. Enrichment of 5hmC is linked to highly expressed genes [10, 11] in the absence of 5mC, suggesting that, in the context of this binding interaction, MeCP2 facilitates transcription [11]. Of note, MeCP2 is itself subject to methylation-dependent regulation, disruptions which have been linked to autism [12]. Thus, epigenetic modifications can regulate both the expression of MeCP2 and its downstream binding partners.

Alternative splicing of* Mecp2*/*MECP2* generates two main isoforms that differ exclusively at the N-terminus [13, 14]: MeCP2_e1, the predominant isoform in the brain [13–16], and MeCP2_e2, which displays a later expression onset during mouse brain development [17]. The two isoforms exhibit differential temporal and region-specific differences in their expression profiles in the brain and both contribute to neurological function and gene expression patterns [18–20]. The ratio of splice variants differs in a temporal- and cell type-specific manner, suggesting dynamic regulation of their expression and nonredundant functionality in the distinct stages of neurogenesis and adulthood [15, 17, 18, 21]. Whereas MeCP2_e1 has been shown to be the isoform most relevant to RTT pathogenesis [20], MeCP2_e2 interacts with forkhead protein FoxG1, which promotes neuronal survival and maturation and in which mutations can also cause RTT [22]. The physiological significance of these two isoforms is not fully understood. Manipulations of independent isoforms in a cell type-specific manner are required in order to reveal their respective contributions to activity-dependent functions of MeCP2.

MeCP2 expression affects successive stages of brain development including prenatal neurogenesis, postnatal development of synaptic connections and function, experience-dependent synaptic plasticity, and maintenance of adult neural function including sensory integration [1, 23, 24]. MeCP2 critically maintains synaptic excitation (E) and inhibition (I), which are fundamental to the function of brain circuits and are often disrupted in neurological disorders including RTT [23, 25]. Additionally, MeCP2 has a remarkably diverse pool of binding partners and downstream targets [26, 27]. This functional and binding complexity, in combination with the domain-specific functionality of the MeCP2 protein [28, 29], confers a pleiotropic effect across age- and cell type-specific backgrounds [7]. Accordingly, different mutations in MeCP2 result in a wide range of phenotypic variability and severity in RTT patients [30], necessitating context-dependent mechanistic insights into MeCP2 function.

Transgenic mouse models that harbor cell type-specific mutations in MeCP2 have shed light on our understanding of RTT pathogenesis in the brain. Expression of MeCP2 under the CamKII or neuron-specific enolase promoter does not prevent the appearance of most RTT phenotypes, suggesting a more complex network of involvement for MeCP2 [31]. Interestingly, mice lacking MeCP2 exclusively in GABAergic neurons recapitulate many RTT features [32], and deletion of MeCP2 in the parvalbumin- (PV-) expressing subset of GABAergic neurons abolishes experience-dependent critical period plasticity in the absence of most RTT phenotypes [33]. The restoration of MeCP2 exclusively in astrocytes results in a non-cell-autonomous ameliorative effect on neurons* in vivo* [34], whereas RTT microglia exhibit adverse non-cell-autonomous effects on WT neurons* in vitro* [35]. In spite of their differing roles and effects on downstream gene regulation [18], transgenic expression of either the MeCP2_e1 or MeCP2_e2 splice variant has been shown to prevent the development of a number of RTT phenotypes in a mouse model lacking MeCP2. However, many abnormalities were only partially prevented, negating the notion that both transcripts are capable of acting independently to fulfill all of the roles of MeCP2 [36]. Accordingly, another study demonstrated that a point mutation in the MeCP2_e1 splice variant is sufficient to recapitulate many RTT phenotypes observed in MeCP2 KO mice [20]. Whereas the complexity that underlies the roles of MeCP2 will not be resolved with a single mouse model, each contributes a piece to the larger puzzle that represents MeCP2 functionality.

Similar to loss-of-function mutations in* MECP2*, the duplication of* MECP2* also results in a progressive neurological disorder that includes stereotypic and repetitive hand or body movements, epilepsy, spasticity, and a severe syndromic form of intellectual disability in male patients [37]. Recent studies show that the neurological dysfunctions in* MECP2* duplication syndrome are reversible in adult symptomatic mice and correction of MeCP2 levels genetically or by using antisense oligonucleotides largely restores molecular, electrophysiological, and behavioral deficits [38].

**Figure 1:**
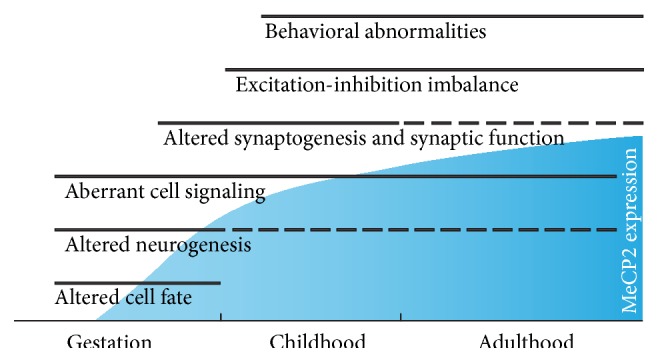
MeCP2 influences multiple features of brain development and function, at a variety of time points as its expression increases and is maintained. Thus, prenatal and postnatal brain development, as well as adult function, are all potentially affected in Rett Syndrome.

Traditionally, the dynamic time-course of RTT is thought to involve a period of apparently normal early development followed by profound neurological regression—a defining feature of RTT—and subsequent stabilization or partial recovery. However, our understanding of the disease initiation and progression and the ways in which* MECP2* impacts distinct phases of neurodevelopment is gradually evolving. In recent years, there has been a gradual shift in our understanding of atypical regression in RTT patients, with growing evidence of prenatal and early postnatal developmental abnormalities resulting in defects in the establishment and refinement of early neural circuits and, later, cortical plasticity (Figure 1). In this review, we aim to summarize recent findings and argue that* MECP2* serves distinct, discrete functions throughout developmental and adult stages, integrating genomic and environmental signals in a context-dependent manner.

## 2. Deficits in Early Neurogenesis

Early work in mouse models on the function of MeCP2 reported a pattern of expression limited to the neural lineage, with low expression in neuroblasts and a progressive increase during embryonic and postnatal development. Such findings led to the belief that MeCP2 is predominantly involved in the maturation and maintenance of neuronal function, as opposed to early cell fate decisions, and were further supported by a lack of phenotype observed with respect to differentiation in MeCP2-null neural progenitor cells (NPCs) [39].

Evidence has since demonstrated that, whereas MeCP2 expression increases postmitotically, both mRNA and protein can be detected throughout the majority of the mouse and human lifespan, including embryonic stages during which neurogenesis occurs [21, 40–44]. MeCP2 protein expression does indeed increase after neuronal differentiation, when the vast majority of RTT phenotypes have been described. Experiments designed to determine isoform-specific expression have detected MeCP2_e1 protein in the mouse hippocampus as early as E14, whereas MeCP2_e2 was first detected at E18 [17]. Samples younger than E14 were not analyzed in this study, and thus these results do not preclude the possibility of expression at earlier embryonic stages. MeCP2_e1 expression increased until it reached a plateau at P7–P21. MeCP2_e2 expression overlapped with MeCP2_e1 after E18, albeit at a decreased level. As the gestational period of synaptogenesis overlaps with that of neuronal migration in human development [45], it stands to reason that MeCP2, known to regulate synaptic development [46–48] and cell guidance and laminar organization in the olfactory system [49], may contribute to the processes of cell fate specification and migration in the developing brain, especially the cortex. Accordingly, the landscape of clinical literature has been shifting to suggest an earlier onset of symptoms in RTT patients [50–54].

## 3. Cell Fate and Signaling Pathways

Expression of MeCP2 during early neurogenesis suggests a consequent role for the protein during this critical developmental time point. Neurogenic functions of MeCP2 have indeed since been demonstrated in mouse, whereby embryonic NPCs overexpressing MeCP2 exhibited a heightened neural identity* in vitro* [55]. Conversely, embryonic NPCs extracted from mice lacking MeCP2 exhibited a more proliferative—as opposed to late postmitotic—identity and revealed morphological alterations as early as 3 days* in vitro* (DIV) [44]. A human patient-derived induced pluripotent stem cell (iPSC) model of Rett Syndrome expressing a* de novo* frame-shift mutation in exon 4 (c.806delG) illustrated a parallel role for MeCP2 in the promotion of neural identity in which neural stem cells lacking MeCP2 exhibited increased astrocytic differentiation* in vitro* [56]. Mesenchymal stem cells (MSCs) isolated from a Rett patient harboring a different* de novo* mutation (del 1164-1207) also demonstrated impaired neural differentiation* in vitro*, which resulted in a reduced percentage of NeuN-expressing cells and increased senescence [57]. Roles for MeCP2 in determining neurogenic potential have been reported in* Xenopus* [58], zebrafish [59, 60], and chick [61] embryos.

Mechanisms underlying the function of MeCP2 with respect to early cell fate decisions are largely unknown. Neurogenic signaling cascades such as the* Notch-Delta* and PI3K-Akt pathways have been demonstrated to coordinate with MeCP2 throughout various time points including neurogenesis. Phosphorylation of MeCP2 at Serine421 (S421)—known to regulate gene transcription and synaptic development in an activity-dependent manner [62]—has since been shown to modulate the balance between proliferation and differentiation in NPCs isolated from the adult mouse hippocampus. Evidence suggests that the* Notch-Delta* signaling pathway, mediated via MeCP2-S421 phosphorylation, may serve as the hub linking MeCP2 to neural cell fate decisions in adult NPCs [63]. Experiments performed in* Xenopus* embryos, in which MeCP2 is expressed and is critical for neurogenesis, have demonstrated that the* Notch-Delta* signaling pathway regulates the patterning of primary neuronal differentiation in conjunction with MeCP2 binding. A complete lack of MeCP2 protein resulted in a decrease in the number of neuronal precursors, whereas expression of a truncated form of MeCP2 often found in Rett Syndrome patients—R168X—resulted in an increase in the number of neuronal precursors relative to WT embryos [58]. This phenotypic variety observed as a result of varying dosages and mutations of MeCP2 is echoed throughout the experimental and clinical literature [30, 52, 53, 64].

The PI3K-Akt signaling pathway is implicated in a wide range of cellular functions including cell cycle and transcriptional regulation [65]. The pathway has also been shown to regulate key neurological processes such as synaptic transmission [66] and neurodegeneration [67, 68] and is implicated in a range of neurological diseases and disorders such as spinocerebellar ataxia type 1 [67], Huntington's disease [68], amyotrophic lateral sclerosis (ALS) [69], and RTT [4, 70–72]. The majority of studies performed in RTT models, including those listed above, have examined the contribution of the PI3K-Akt pathway to disease effects and rescue in mature neurons. Whereas PI3K-Akt signaling has been shown to promote adult neurogenesis in the context of exercise enrichment [73], traumatic brain injury recovery [74], and surgical denervation [75], roles for PI3K-Akt signaling have also been demonstrated throughout embryonic neurogenesis in mouse [76],* Xenopus* [77], and zebrafish [78]. However, the precise roles of PI3K-Akt signaling in embryonic neurogenesis in the context of RTT have yet to be elucidated.

## 4. microRNAs and MeCP2

microRNAs (miRNAs) finely regulate genetic networks throughout the course of brain development and, with astounding complexity, act as critical determinants of early neurogenic activities such as cortical patterning and activity development, cellular subtype specification, and neuronal differentiation [79–81]. They are themselves subject to upstream epigenetic regulation; many are indeed targets of MeCP2 [82] or, as in the case of miR-132, act in a feedback loop as both target and regulator to maintain MeCP2 levels [83]. miR-132 has in turn been shown to promote postnatal neurogenesis and synaptic integration in neurons of the olfactory bulb [84]. Another brain-enriched miRNA target of MeCP2, miR-137, has been shown to regulate neuronal maturation and dendritogenesis in the postnatal hippocampus [85] and to modulate proliferation and differentiation in adult neurogenesis [82]. Moreover, miR-137 has been shown to negatively regulate neural stem cell proliferation and promote differentiation in the embryonic mouse brain [86]. miR-199a has been demonstrated as a link between MeCP2 and the mTOR pathway [87], previously implicated in RTT [72]. MeCP2 facilitates the postprocessing of miR-199a, which positively regulates mTOR signaling. Notably, exogenous miR-199a ameliorates several impairments in RTT neurons and the genetic deletion of miR-199a-2 results in decreased mTOR activity in the brain and the recapitulation of several RTT phenotypes [87]. MeCP2 is known to influence the production of growth factors such as BDNF and IGF1—the latter via a miRNA-mediated pathway downstream of BDNF [88]. Many pathways and loops that determine the process of neurogenesis are maintained by the concerted regulation of miRNAs and MeCP2. As such, they provide insight into potential avenues by which MeCP2—or the lack thereof—can influence the developing cortex.

## 5. Deficits in Neuronal Migration and Cortical Patterning

Functions of MeCP2 during early neurogenesis result in immediate and long-term effects on neuronal migration and cortical patterning. Migration begins at gestational week 8 in humans and at E11 in mouse, at which point neural progenitors proliferating within the ventricular zone that lines the cerebral ventricles begin to differentiate to form the cortical laminae [45, 89]. Postmitotic cells migrate over radial glial scaffolds to form the discrete layers of the cerebral cortex in an inside-first, outside-last temporal pattern. Deep-layer cortical neurons are born first and passed by newly born neurons migrating to upper layers. This process is spatiotemporally governed by a variety of signaling, transcriptional, and epigenetic mechanisms [90–92]. Aberrant regulation of the proliferation and differentiation of neural stem cells results in a range of cortical dysplasias and is associated with many neurological and neuropsychiatric disorders including Alzheimer's disease, schizophrenia, and ASDs [93].

Early work demonstrated morphological cortical deficits in 8-week-old MeCP2^−/Y^ mice including reduced thickness and increased cell density in neocortical layers; due in part to the belief that MeCP2 was not expressed early on, these alterations were believed to be a result of reduced cell size and complexity as opposed to deficits in corticogenesis [39]. Cerebellar expression profiling performed alongside chromatin immunoprecipitation in MeCP2-deficient mice has since revealed increased expression of* Reln*—encoding the extracellular signaling protein Reelin, known to be essential for proper neuronal lamination [94]. Accordingly, recent evidence has demonstrated that mouse NPCs lacking MeCP2 exhibit delayed corticogenesis with respect to migration from the subventricular and ventricular zones into the cortical plate [44]. These findings suggest a need for a thorough evaluation of the role of MeCP2 in cortical migration and lamination, as layering deficits observed at postnatal time points in RTT may result from combinatorial deficits in cortical development and maintenance.

## 6. Deficits in Synaptic Transmission and Plasticity during Postnatal Development

Along with deficits in early neurogenesis and cortical patterning,* MECP2* has been shown to play a key role in synaptic maturation and plasticity. Mutant mouse models have been generated with a global deletion of MeCP2 (MeCP2^−/Y^) from all neurons and selectively from specific cellular subtypes including various neuronal subtypes and astrocytes. These models have served as a robust starting point in which to study the common principles underlying synaptic defects in RTT. They provide unique insight into the genetics that determine cell type-specific contributions to pathogenesis.

Functional defects in synaptic transmission have been investigated in an* Mecp2* global deletion model in which cortical connections were found to have weaker excitatory synaptic transmission and lower levels of basal activity [1, 95–98], reminiscent of an immature circuit. Cellular mechanisms of long-term plasticity, considered the functional basis of learning and memory, have also been found to be impaired in* Mecp2* mutant animals [2, 99, 100]. The majority of these early studies have used brain slice preparations, recording synaptic transmission including miniature synaptic currents and synaptic plasticity deficits. Similar to deficits in excitatory transmission, deletion of* Mecp2* from all forebrain GABAergic interneurons also recapitulates key features of RTT, suggesting that inhibition plays a crucial role in RTT pathophysiology. This includes reduced GABA synthesis, Gad1 and Gad2 levels, reduced miniature inhibitory postsynaptic currents (mIPSCs), and an array of behavioral deficits including EEG hyperexcitability and severe respiratory dysrhythmias [3, 4, 32]. Anatomical studies have reported enhanced PV+ neuronal puncta and hyperconnected PV+ circuitry in mouse visual cortex, suggesting that these microcircuits contribute to enhanced inhibition in MeCP2^−/Y^ mice [5, 101]. This altered inhibition mediated by PV+ neurons, which regulates the initiation and termination of the critical period, has been proposed to alter the timing of critical period plasticity in RTT [6, 102]. Functional studies, however, have consistently reported decreased inhibitory function including reduced mIPSCs in CA3 pyramidal neurons of MeCP2^−/Y^ mice [3, 7, 8, 103]. Although the density and intrinsic membrane properties of PV+ and somatostatin (SST)+ interneurons were not affected in MeCP2^−/Y^ mice, miniature excitatory postsynaptic currents (mEPSCs) were found to be smaller and less frequent in fast-spiking PV+ neurons, suggesting impaired glutamatergic drive specifically onto this interneuron population compared to SST+ neurons [103]. Studies in slices have also reported a reduction in mEPSC amplitudes and a deficit in excitatory pathways, in the absence of change in mIPSC amplitude or frequency [95, 104]. These results are consistent with the decreased visually evoked responses found in PV+ interneurons in mouse visual cortex* in vivo* [33]. Interestingly, recent studies have highlighted the differential effects of subtype-specific* Mecp2* deletion on GABAergic inhibition regulating nonoverlapping neurological symptoms: mice lacking MeCP2 in PV+ neurons showed sensory, motor, memory, and social deficits, whereas those lacking in SOM+ neurons exhibited seizures and stereotypies [105], further elucidating the complex regulation of inhibition and their disruption in RTT [23].

Taken together, these features indicate that RTT is a complex disorder that arises from an imbalance of excitation and inhibition and a failure of brain circuitry to attain a mature state [9, 23]. Many of these defects can have a strong early developmental, even prenatal, component (Figure 1) when the brain fails to attain “phenotypic checkpoint” signatures and in turn provides faulty functional feedback that influences gene expression [106] and network malfunction [107]. A coherent set of physiological measurements using* in vivo* awake animal models of global and neuronal subtype-specific* Mecp2* deletion remains necessary to measure and evaluate functional defects in the synaptic balance of excitation and inhibition. Another important consideration in this regard is to extend findings of cell-specific and synaptic defects in mouse models to identify biomarkers of RTT in human patients. Several recent studies are bridging this gap. Visual evoked potential (VEP) recordings in response to high-contrast oriented gratings have previously revealed loss of visual acuity in adult* Mecp2* mutant mice at the onset of RTT-like symptoms during critical periods of mouse visual cortex development [101], strongly suggesting that vision may serve as a biomarker of altered cortical function in RTT. Recent work has demonstrated that RTT patients exhibit a similar decrease in VEP amplitude and a reduction in visual spatial acuity that is impacted by* MECP2* mutation type [108].

## 7. Deficits in Adult Maintenance and Function

The onset of symptoms during early life in RTT patients, in conjunction with findings from mouse models suggesting neurodevelopmental abnormalities in RTT, has raised the question whether* Mecp2* function is necessary for integrative function in the adult brain. One study used an inducible knockout approach to delete* Mecp2* by crossing a floxed* Mecp2* allele mice with a tamoxifen-inducible Cre-ER expressing allele in adult mice (P60 or older) following normal development [109]. This late-deletion of* Mecp2* recapitulated key germline knockout phenotypes including abnormal gait, hind-limb clasping, motor abnormalities, impaired nesting ability, and impaired learning and memory, further underscoring the importance of* Mecp2* in adult neurological function [109]. Interestingly, this adult deletion recapitulated an epigenetic memory clock, suggesting a mechanism that extends—or is independent from—its early global genetic regulation [110].

Similar to behavioral deficits, the physiological response features of adult* Mecp2*-deleted neurons have also been characterized* in vivo* [111]. CRISPR-associated endonuclease (Cas) 9 has been used to introduce frame-shifting, insertion/deletion (INDEL) mutations that are targeted to the* Mecp2* locus using specific guide RNAs (gRNAs) via adeno-associated viral (AAV) vectors [112].* In vivo* genome-editing resulted in ~68% of targeted cells containing INDEL mutations with a >60% reduction in MeCP2 protein levels [111]. Stereotactic injection of AAV-SpCas9 and gRNA targeting* Mecp2* into the superficial layers of mouse primary visual cortex followed by two-photon guided targeted electrophysiological recordings from genome-edited neurons revealed altered integrative visual responses, further emphasizing the maintenance role of* Mecp2* in the adult brain after normal developmental milestones have been achieved.

## 8. Reversal of Functional and Behavioral Deficits in RTT

One of the key discoveries in RTT has been the recovery of function following reactivation of endogenous* Mecp2* [113, 114]. This striking finding, an important feature not only of RTT but perhaps also of neurodevelopmental disorders in general, suggests that the neurodevelopmental pathology is reversible.

The phenotypic reversibility of advanced neurological phenotypes in both immature and mature adult animals shows that reactivation of the MeCP2 protein even at late stages of the disorder can partially rescue the mutant phenotype [113, 115]. Systemic delivery of MeCP2 cDNA via AAV9, under control of a fragment of its own promoter (scAAV9/MeCP2), has been shown to significantly rescue behavioral and cellular deficits when administered systemically into female RTT mice [116]. Proposed as a model for gene therapy, the retroviral-mediated overexpression of the MeCP2_e1 isoform in neural stem cells taken from* Mecp2* heterozygous mice was shown to promote dendritic branching* in vitro* [117]. Perhaps more practically, pharmacological manipulations, such as the treatment of* Mecp2* null mice with recombinant human IGF1 (rhIGF1) or a peptide fragment of IGF1, also resulted in a partial rescue of synaptic defects and cortical excitatory synaptic transmission, in addition to restoring activation of signaling pathway proteins [70, 71]. These studies argue that the brain circuits involved in neural processing may not functionally decline but rather remain in a labile, immature state; their subsequent activation by the reintroduction of* Mecp2* [113, 115] or by pharmacological manipulations to activate downstream signaling pathways [70, 71] is an important measure to ameliorate the syndrome's consequences.

## 9. Conclusions

The fluidity with which MeCP2 regulates the genomic landscape renders a uniquely moving target that has proven difficult to fully understand. Amongst many factors to be taken into account when attempting to attribute mechanistic function to MeCP2 (e.g., cell type, mutation and associated functional domain, and range of downstream targets), it is crucial to consider the time point in question. Deletion of MeCP2 results in a wide and temporally varied range of phenotypes. A complete picture of the MeCP2 protein includes its roles at various life stages, so as to inform our evolving concept of RTT progression in patients and potential phenotypic reversibility.
